# Preparation and Electrical Properties of Silicone Composite Films Based on Silver Nanoparticle Decorated Multi-Walled Carbon Nanotubes

**DOI:** 10.3390/ma14040948

**Published:** 2021-02-17

**Authors:** Kwang Se Lee, Isheunesu Phiri, Sang Hern Kim, Kyeongkeun Oh, Jang Myoun Ko

**Affiliations:** 1 Department of Advanced Materials & Chemical Engineering, Kyungnam College of Information & Technology, 45 Jurye-ro, Sasang-gu, Busan 47011, Korea; inraining82@gmail.com; 2 Department of Chemical and Biological Engineering, Hanbat National University, 125 Dongseo‐daero, Yuseong‐gu, Daejeon 34158, Korea; isheunesuphiri@gmail.com (I.P.); shkim@hanbat.ac.kr (S.H.K.); 3 Institute for New Technology Education, Korea Polytechnics, 20 Yusang-ro, Deokjin-gu, Jeonju 54853, Korea

**Keywords:** multi-walled carbon nanotube, acylation, modification, film, electrical conductivity

## Abstract

The electrical properties of silicone composite films filled with silver (Ag) nanoparticle-decorated multi-walled carbon nanotubes (MWNT) prepared by solution processing are investigated. Pristine MWNT is oxidized and converted to the acyl chloride-functionalized MWNT using thionyl chloride, which is subsequently reacted with amine-terminated poly(dimethylsiloxane) (APDMS). Thereafter, APDMS-modified MWNT are decorated with Ag nanoparticles and then reacted with a poly(dimethylsiloxane) solution to form Ag-decorated MWNT silicone (Ag-decorated MWNT-APDMS/Silicone) composite. The morphological differences of the silicone composites containing Ag-decorated MWNT and APDMS-modified MWNT are observed by transmission electron microscopy (TEM) and the surface conductivities are measured by the four-probe method. Ag-decorated MWNT-APDMS/Silicone composite films show higher surface electrical conductivity than MWNT/silicone composite films. This shows that the electrical properties of Ag-decorated MWNT-APDMS/silicone composite films can be improved by the surface modification of MWNT with APDMS and Ag nanoparticles, thereby expanding their applications.

## 1. Introduction

Carbon nanotubes (CNTs) are formed from concentrically-rolled graphene sheets with asymmetric helicity instead of the initially proposed scroll-like roll, therefore, CNTs have special mechanical, electrical, thermal, and optical properties [1,2,3,4,5]. The sp^2^ carbon atoms in the carbon skeleton provide excellent high electronic and thermal conductivity, and chemical stability [6]. Compared to other conducting carbons, CNTs have a high aspect ratio and high specific surface area, therefore only a small amount can be used in forming a conductive path in composites. Multi-walled carbon nanotubes (MWNT)-hybridized metal nanoparticles have recently received extensive attention including single-electron transistors, molecular diodes, memory elements, and logic gates [7,8,9,10,11,12,13,14]. Among them, Ag-decorated CNTs especially gained attention owing to their potential applications as optical limiters and advanced materials [15,16,17]. However, CNTs do not disperse well in most organic solvents, resulting in poor homogeneity when mixed with a polymer matrix [18]. One way to improve the dispersibility of CNTs in organic solvents is to modify the surface of the CNTs by introducing functional groups that enhance interaction with the solvents [19,20,21]. Before any modification, the CNTs are first purified by acid treatment, where impurities are dissolved leaving the CNT surface-functionalized with carboxylic acid groups. However, the acid treatment damages the CNT causing a reduction in electrical conductivity of the CNT [19,20,21,22]. Since defective sites inevitably accompany modification of CNT, acid treatment conditions need to be optimized to minimize the damage on the CNT, thereby minimizing the reduction in electrical conductivity. In this study, MWNT were modified by an acylation reaction [22] and consequently coated with silicone oil under sonication. Then, silicone-modified MWNT was reacted with Ag nitrate, based on the wet chemical reaction, to form Ag-decorated MWNT. The Ag-decorated MWNT was employed as a conductive filler material in a silicone matrix to get a composite film with higher electrical conductivity. The as-prepared Ag-decorated MWNT silicone composite has the potential to be used as a conductive filler in the electrical packaging industry.

## 2. Materials and Methods

### 2.1. Materials

The MWNT (>95%, length = 10–50 μm; diameter = 10–20 nm) were supplied by Hanwha Nanotech Co., Seoul, Korea. Thionyl chloride (Samchun Chemical Co., Seoul, Korea) was used as a reacting agent without any purification. Tetrahydrofuran (THF, Duksan Chemical Co., Ansan, Korea) was used for dissolving amino terminated poly(dimethylsiloxane) (APDMS, Functional group equivalent: 2200 g/mol, Shin-Etsu Co., Tokyo, Japan). *N*-methyl-2-pyrrolidone (NMP, Samchun Chemical Co., Seoul, Korea) was used as a reducing agent to reduce silver nitrate (Sigma Aldrich., Co., St. Louis, MO, USA) to Ag nanoparticles. Poly(dimethylsiloxane) (PDMS, Momentive Performance Materials Co., Waterford, NY, USA) dissolved in THF with a functional group equivalent of 2200 g/mol was used as the silicone matrix.

### 2.2. Synthesis of Ag Nanoparticle Decorated MWNT

A total of 1 g of the MWNT was dispersed in a mixture of HNO_3_ and H_2_SO_4_ (1:3 *v*/*v*) under mechanical stirring for 2 h at 80 °C. The mixture was filtered and washed with distilled water until pH 7, and dried at 80 °C for 24 h in an oven (Fisher Scientific, Pittsburgh, PA, USA). The acid-treated MWNT (MWNT-COOH) was reacted with excess thionyl chloride under reflux for 24 h to obtain an acyl chloride-functionalized MWNT (MWNT-COCl). After filtering and subsequent washing with distilled water until pH 7, 0.05 g of the MWNT-COCl powder was suspended in 100 mL solution of APDMS under sonication to form amide functionalized MWNT (MWNT-APDMS). Excess APDMS was removed by sonicating the MWNT-APDMS in THF and then filtered and dried. The silicone layer on the MWNT-APDMS surface was cured at 270 °C (the curing temperature was determined using DSC). To decorate the MWNT-APDMS with Ag nanoparticles, the MWNT-APDMS were dispersed in a silver nitrate solution (0.4 M in NMP) using a bar sonicator (Fisher Scientific, Pittsburgh, PA, USA) for 3 min and then mechanically stirred for 60 min at 140 °C. The Ag-decorated MWNT-APDMS were filtered and repeatedly centrifuged 3 times at 1000 rpm for 10 min using NMP as a dispersant. The precipitate was dried at 230 °C in a furnace. The same procedure was followed for decorating MWNT-COOH with Ag nanoparticles.

### 2.3. Sample Preparation

The Ag-decorated MWNT-APDMS were suspended in PDMS/THF solution (1:6 *v*/*v*) under sonication in an ice bath for 10 min. The Ag-decorated MWNT-APDMS/Silicone composite films were then coated on polyethylene terephthalate (PET) by dispersing 20 mL of the mixture uniformly onto a 15 mm × 15 cm PET film before curing at 80 °C for 1 h and at 150 °C for 3 h in an oven.

### 2.4. Measurements

The infrared spectra were measured using a Fourier Transform Infrared Spectrometer (FT-IR, FTS-60, Bio-Rad Co., Philadelphia, PA, USA). A Transmission Electron Microscope (TEM, Tecnai 20, Eindhoven, Netherlands) was used to determine the morphologies at an accelerating voltage of 200 kV. Scanning Electron Microscopy images were taken using a Field Emission Scanning Electron Microscopy (FESEM, Hitachi S-4300, Tokyo, Japan). Thermal stability was determined using Thermogravimetric analysis (TGA, Q50, TA instrument Co., New Castle, DE, USA) at a rate of 10 °C/min, a temperature range of 25–100 °C in air atmosphere. The electrical conductivity was evaluated according to the four-probe method (Loresta-GP, Mitsubishi Chemical Co., Tokyo, Japan).

## 3. Results and Discussion

**Figure 1:**
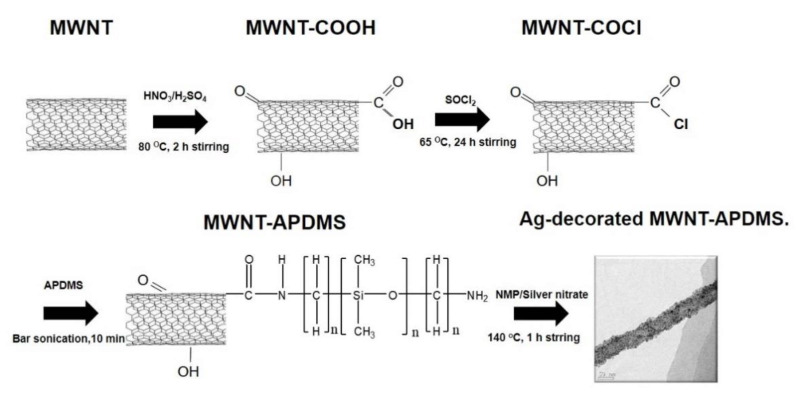
Schematic procedure for the preparation of multi-walled carbon nanotubes-amine-terminated poly(dimethylsiloxane) (MWNT-APDMS).

**Figure 2:**
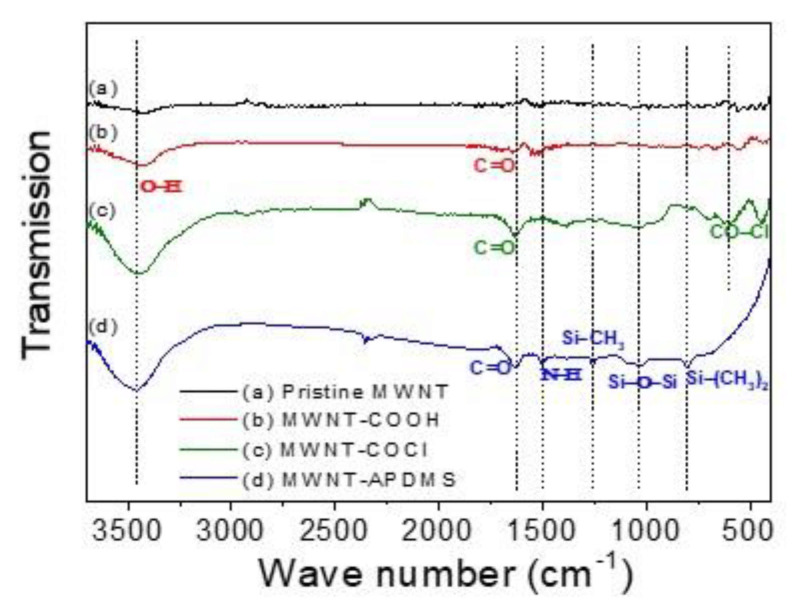
FT–IR spectra of the (**a**) pristine MWNT, (**b**) MWNT–COOH, (**c**) MWNT–COCl, and (**d**) MWNT–APDMS.

Figure 1 illustrates the modification of the MWNT and Figure 2 shows the FT-IR spectrum of pristine MWNT, MWNT-COOH, MWNT-COCl, and MWNT-APDMS. In comparison with pristine MWNT, the characteristic peaks in Figure 2b at 1620~1640 cm^−1^ and 3300–3600 cm^−1^ are attributed to the C=O stretching vibration and O–H stretching vibration of the carboxylic groups derived from acid treatment [22]. This shows that the MWNT was successfully oxidized by the acid mixture to form MWNT-COOH. In Figure 2c, the peak at 430 cm^−1^ is due to the –C(O) –Cl stretching vibration formed when the MWNT-COOH were converted to MWNT-COCl by reacting with thionyl chloride [22]. In Figure 2d, the peaks at 800~820 and 1000~1200 cm^−1^ are attributed to stretching vibration of Si–(CH_3_)_2_ and Si–O–Si, respectively [23]. Furthermore, the Si–CH_3_ stretching vibration at 1250~1300 cm^−1^ is also observed [23]. Additionally, peaks at 1620~1640 and 1500–1550 cm^−1^ due to the C=O stretching vibrations and N–H bending vibrations were observed in the spectrum corresponding to amide bonding [22]. The existence of these characteristic peaks gave direct proof for the covalent attachment.

**Figure 3:**
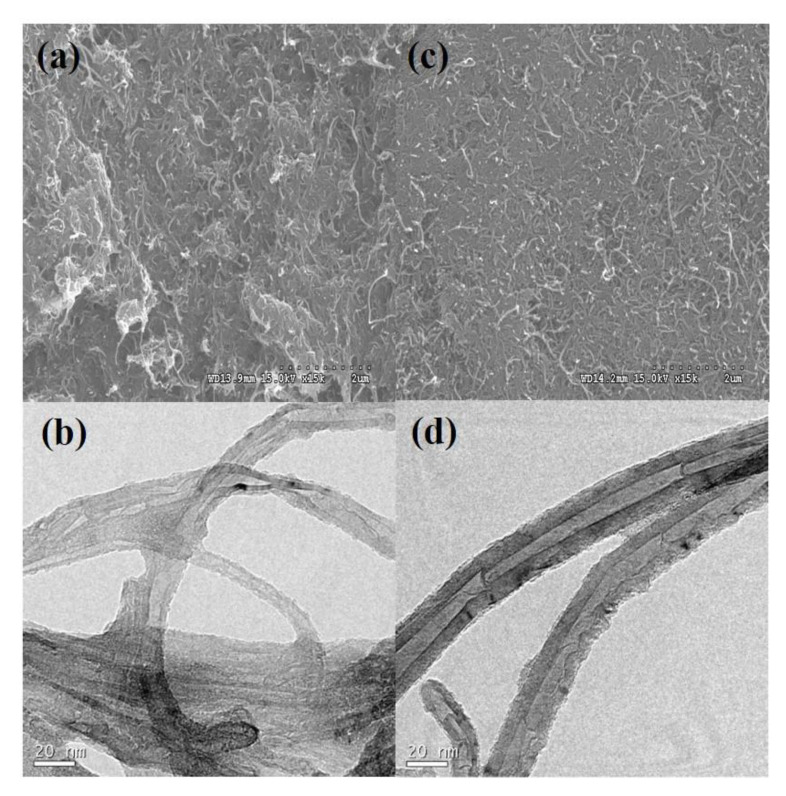
FESEM images of MWNT–APDMS with (**a**) and (**b**) before washing excess APDMS, (**c**), and (**d**) after washing excess APDMS.

Figure 3a shows an FESEM image of the MWNT–APDMS before washing with the THF solvent. The APDMS were agglomerated owing to excess APDMS which is further revealed by the TEM image in Figure 3b. However, there is a reduction in agglomeration after removal of excess APDMS by THF solvent as shown by both the SEM and TEM images (Figure 3c,d). After washing off the excess APDMS, the APDMS was neatly decorated on the surface of the MWNT.

**Figure 4:**
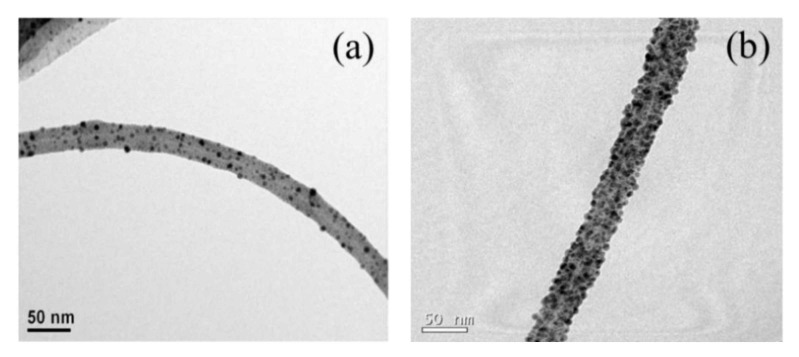
TEM images for (**a**) Ag–decorated MWNT–COOH and (**b**) Ag–decorated MWNT–APDMS. The average size of silver nanoparticles is ~8 nm.

Both the MWNT–COOH and MWNT–APDMS were decorated with Ag nanoparticles for comparison and their TEM images are shown in Figure 4. Figure 4a shows the MWNT–COOH decorated with Ag nanoparticles which appear as spherical black dots, however, the TEM image for the Ag–decorated MWNT–APDMS (Figure 4b) shows more Ag nanoparticles homogeneously dispersed and strongly attached on the MWNT surface because the terminal–NH_2_ functional group forms stronger attractive forces with the Ag nanoparticles. This happens because of the coordinate bonding between the Ag^+^ ions and amine groups resulting in monodispersive attachment of Ag nanoparticles on the MWNT surface [24].

**Figure 5:**
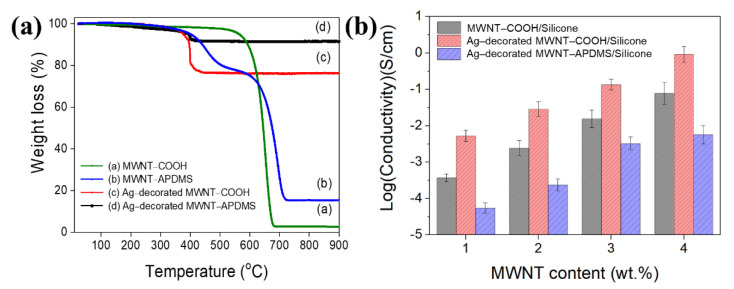
(**a**) TGA analysis curves of the samples and (**b**) The surface conductivity of silicone composite films depending on MWNT content.

Figure 5a shows the thermal stability of MWNT–COOH, MWNT–APDMS, Ag–decorated MWNT–COOH, and Ag–decorated MWNT–APDMS. The MWNT–COOH is stable up to ~517.5 °C where it rapidly decomposes until there is ~2.4 wt.% of the residue left at ~681.3 °C. The residue is due to the presence of metallic catalysts used during the production of MWNT by the catalytic chemical vapor deposition method [25,26,27]. The MWNT–APDMS shows a two–stage thermal decomposition profile. The first stage starts at ~390.7 °C up to ~681.3 °C, then the second stage begins and rapid decomposition follows until there is ~15.5 wt.% of the residue left at ~721.3 °C. The first decomposition stage happens because, as temperature increases, condensed phase oxidation of APDMS happens that leads to further tight crosslinking of the polymer, thereby enhancing its thermal stability. As the temperature continues to rise, the tightly cross–linked polymer starts to decompose leaving silica residue from the siloxane backbone and the metallic catalysts used in synthesizing the MWNT leading to an increased residue weight (~15.5 wt.%) [28]. From this, it can be inferred that the composition of APDMS is ~13.1 wt.%. The Ag–decorated MWNT–COOH were stable up to ~390.7 °C and started to rapidly decompose up to ~440.8 °C, the same trend was observed for the Ag–decorated MWNT–APDMS, however, the former had a ~76.5 wt.% and the latter ~91.5 wt.% of residue left owing to the greater percentage composition of the Ag nanoparticles in the Ag–decorated MWNT–APDMS.

The surface electrical conductivities were done for silicone composite films containing 1–4 wt.% of each of MWNT–COOH, Ag–decorated MWNT–COOH, and Ag–decorated MWNT–APDMS (Figure 4b). It was expected that the Ag nanoparticles would enhance the electrical conductivity of MWNT because Ag is inherently electrically conducting. The conductivity (σ) of the composite films was calculated using Equation (1).

(1) $$\sigma =\frac{T}{LW}\left(\frac{1}{R}\right)$$

where the *T*, *L*, and *W* indicate thickness, length, and width (all in cm) of thin–film, respectively, and R is the resistance (Ω). Generally, as the content of the modified MWNT in the silicone composite increases, the conductivity increases because MWNT are electrically conducting. However, the silicone composite with Ag–decorated MWNTs showed significantly higher conductivities because of the presence of Ag nanoparticles. The silicone composite with the Ag–decorated MWNT–APDMS showed the highest conductivity because it has more Ag nanoparticles than the Ag–decorated MWNT–COOH. The MWNT–APDMS provides a better surface for attachment of amine groups that form coordinate bonding with the Ag nanoparticles [24]. This study shows that the Ag–decorated MWNT–APDMS can be used as a conducting filler in polymer composites with comparable performance as shown in the comparisons table (Table 1).

## 4. Conclusion

This study presents a method to decorate MWNT with Ag nanoparticles and then use them to improve the conductivity of silicone composites. The method included acid treatment of MWNT followed by acylation reaction and grafting of APDMS and thereafter decoration with Ag nanoparticles. Silicone–based composite films were filled with MWNT–COOH, Ag–decorated MWNT–COOH, and Ag–decorated MWNT–APDMS and the electrical conductivity results showed that films filled Ag–decorated MWNT–APDMS had higher conductivity compared to the rest, owing to the large number of Ag nanoparticles attached to the surface of the modified MWNT. This study also shows the practical application of the Ag–decorated MWNT in electronic devices. Among them, our work will play a more positive role in silicon–related battery technology due to the silicone–based synthetic material and excellent affinity with the silicone binder.

## Figures and Tables

**Table 1:** Comparison of electrical conductivity of MWNT/Ag composite materials.

Composite	% of CNT/Ag in Polymer Matrix	Electrical Conductivity (S cm^−1^)	Reference
CNT/Ag in epoxy resin	0.10%	30.53	[25]
CNT/Ag in epoxy resin	65%	102.04	[29]
Ag–CNT/PS (polystyrene)	10	0.65	[30]
Ag@C/MWNT	–	3.85	[31]
Ag/MWNT in polypropylene	3	~4.5 × 10^−4^	[32]
MWNT/PDMS	0	1.83 × 10^−6^	[33]
Ag/MWNT in silicone composite	4%	0.92	This work

## Data Availability

The data presented in this study are available on request from the corresponding author.
